# Animal naming test at discharge is associated with hepatic encephalopathy after elective TIPS

**DOI:** 10.1016/j.jhepr.2025.101701

**Published:** 2025-11-29

**Authors:** Melisande Jorus, Philippe Sultanik, Charlotte Bouzbib, Sarah Mouri, Lyes Kheloufi, Maxime Gasperment, Nicolas Weiss, Charles Roux, Dominique Thabut, Marika Rudler

**Affiliations:** 1 Sorbonne Université, AP-HP, Pitié-Salpêtrière Hospital, Intensive Care Unit, Hepatology Department, Paris, France; 2 Brain Liver Pitié-Salpetrière (BLIPS), France; 3 Sorbonne Université, AP-HP, Pitié-Salpêtrière Hospital, Intensive Care Unit, Neurology Department, Paris, France; 4 INSERM, Centre de Recherche Saint-Antoine (CRSA), Institute of Cardiometabolism and Nutrition (ICAN), Paris, France; 5 Sorbonne Université, AP-HP, Pitié-Salpêtrière Hospital, Interventional radiology, Radiology Department, Paris, France

**Keywords:** Cirrhosis, hepatic encephalopathy, TIPS, animal naming test, ammonia

## Abstract

**Backgrounds & Aims:**

Overt hepatic encephalopathy (OHE) is the most feared complication after transjugular intrahepatic portosystemic shunt (TIPS) placement. We aimed to evaluate the usefulness of the animal naming test (ANT) in predicting the occurrence of OHE after elective TIPS placement.

**Methods:**

The ANT (1 min) was evaluated before TIPS, at discharge, and at 1, 3, and 6 months in all patients treated with elective TIPS between September 2019 and November 2024.

**Results:**

We included 100 consecutive patients (80% men; median age 59 years; median MELD score 11). Indications for TIPS were ascites, secondary prophylaxis, hydrothorax, and pre-surgery in 63%, 19%, 5%, 13%, respectively; 24% had a history of OHE. The median ANT before TIPS was 21 (IQR 17–27). The cumulative incidence of OHE was 30%, considering death and liver transplantation as competing events. In multivariate analysis, independent predictors of OHE development were age (hazard ratio [HR] 1.04; 95% CI 1.00-1.09; *p* = 0.02), pre-TIPS ammonia level (HR 1.01; 95% CI 1.00-1.03; *p* = 0.02), ammonia level at discharge (HR 1.01; 95% CI 1.01-1.03; *p* = 0.04), and ANT at discharge (HR 0.89; 95% CI 0.81-0.97; *p* = 0.005), but not ANT before TIPS. After discharge, the predictive value of ANT was stable for the diagnosis of subsequent OHE.

**Conclusion:**

ANT at discharge may be useful in identifying patients at higher risk of OHE after TIPS.

**Impact and implications:**

Overt hepatic encephalopathy (OHE) remains a major limitation of transjugular intrahepatic portosystemic shunt (TIPS) placement. While the animal naming test (ANT) before TIPS could not accurately predict the development of OHE, both ANT and ammonia after TIPS were predictive of OHE. Each decrease of ANT of 1 point was associated with an increased risk of OHE of 8%. Incorporating this test into routine post-procedural assessment could improve early identification of high-risk patients, guide preventive strategies, and optimize follow-up.

## Highlights

• ANT before TIPS is not predictive of the subsequent development of OHE.
• In this study, both ANT and ammonia after TIPS were predictive of subsequent development of OHE.
• Each 1-point decrease in ANT was associated with an increased risk of OHE of 8%.

## Graphical abstract

**Figure:**
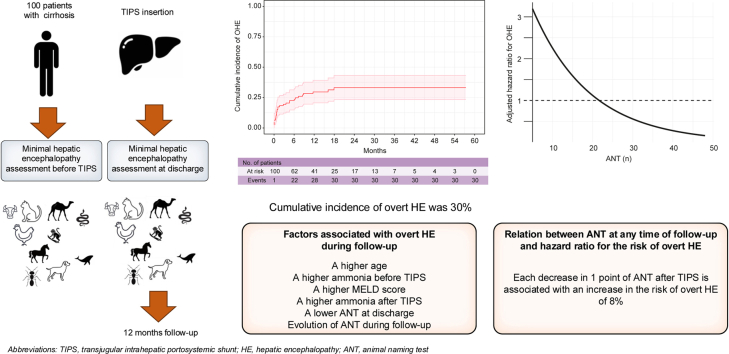

## Introduction

Overt hepatic encephalopathy (OHE) is a significant concern following elective transjugular intrahepatic portosystemic shunt (TIPS) procedures, with studies reporting incidence rates between 30% and 50%, influenced by patient characteristics and the indication for TIPS placement.[1], [2], [3], [4], [5], [6] The impact of OHE on prognosis after TIPS remains a subject of debate. While some studies suggest a negative influence,^6^ others involving a large cohort of over 600 patients indicated that episodic OHE did not elevate mortality risk after elective TIPS.^5^ The timing of OHE onset may also be a factor, with some studies suggesting that early OHE after TIPS could be particularly detrimental.^7^

Predicting neurological outcomes in patients with cirrhosis before elective TIPS is challenging,^8^ but several risk factors for OHE have been identified. These include age, minimal HE (MHE), sarcopenia, a lower portal pressure gradient post-TIPS, renal dysfunction, and hyponatremia, all of which have been associated with a higher likelihood of OHE.^6^^,^[9], [10], [11], [12] Careful patient selection based on individual risk factor assessment, combined with rifaximin prophylaxis in elective settings, represents the most effective approach to preventing OHE after elective TIPS.^13^^,^^14^ The impact of MHE is controversial; some studies show good predictive value for tools such as critical flicker frequency,^12^ whereas others fail to demonstrate any additional predictive value of three tests of MHE before TIPS, including the animal naming test (ANT), the psychometric hepatic encephalopathy score, and the critical flicker frequency.^15^ More recently, one study provided evidence that ammonia levels measured early after TIPS insertion (and before discharge) are an excellent and readily available biomarker for identifying patients at high risk for post-TIPS OHE,^16^ suggesting that the very early consequences of TIPS, more than the baseline characteristics of patients before TIPS, are important in the pathogenesis of post-TIPS OHE. Thus, in this study, we aimed to evaluate whether ANT measured before TIPS and within the first days after elective TIPS could identify patients at high risk of OHE.

## Materials and methods

This is a retrospective study using a prospective cohort of patients treated with TIPS and hospitalized in the Department of Hepatology at La Pitié-Salpêtrière Hospital, Paris, France. This cohort was approved by the research ethics committee of Sorbonne University (CER-2022-074). All consecutive patients treated with TIPS who were hospitalized between September 2019 and September 2024 were screened for inclusion after their non-opposition was recorded in a prospective database. Inclusion criteria were: patients with cirrhosis (previously known or newly diagnosed during hospitalization, confirmed histologically or based on clinical or radiological criteria); elective TIPS placement; and TIPS indications including refractory or recurrent ascites, failure of secondary prophylaxis for acute variceal bleeding, or preoperative management. Exclusion criteria were: age <18; previous liver transplantation (LT); or emergency TIPS (salvage, rescue, or preemptive). ANT was evaluated on the day of TIPS placement, at discharge, and at 1, 3, and 6 months after TIPS insertion during scheduled follow-up visits. Information on the TIPS insertion procedure, follow-up (including clinical evaluation, testing for ANT and OHE, and biological data), and statistics are provided in the supplementary methods. Regarding the use of lactulose or rifaximin as primary or secondary prophylaxis, we applied the following strategy: before the publication of Bureau *et al.*’s study,^13^ all patients with a previous episode of OHE received lactulose as secondary prophylaxis. After the publication of Bureau *et al.*’s study,^13^ all patients with a previous episode of OHE received lactulose as secondary prophylaxis plus rifaximin, starting 15 days before TIPS and continuing for 6 months after TIPS. Also, after Bureau *et al.*’s study,^13^ all patients without a previous episode of OHE received rifaximin, starting 15 days before TIPS and continuing for 6 months after TIPS. During the study period, 270 patients received a TIPS, and 100 patients met the inclusion criteria.

## Results

**Fig. 1:**
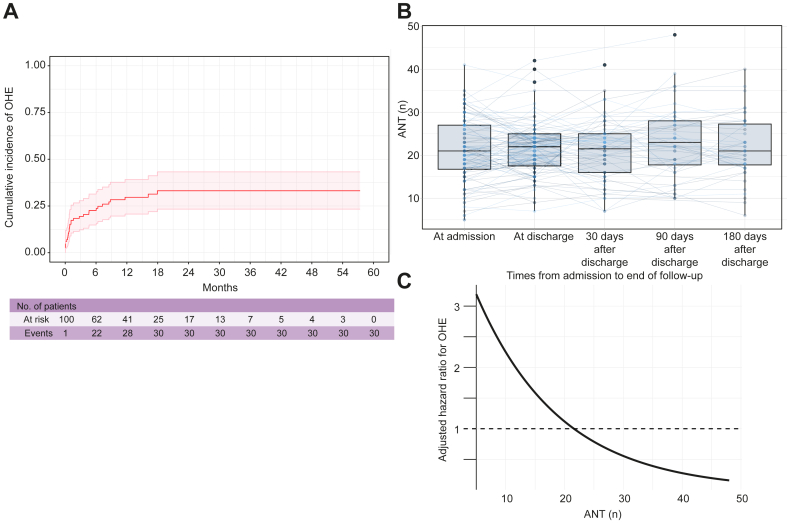
Association between ANT performance and the risk of OHE after TIPS. (A) Cumulative incidence of OHE after TIPS. The cumulative incidence of OHE (considering LT and death as competing risks) was 30%. (B) Trajectory of ANT before TIPS and at discharge, 1, 3 and 6 months after TIPS insertion. (C) Relation between ANT at any time of follow-up and hazard ratio for the risk of OHE. Each decrease in ANT of 1 point after TIPS is associated with an increase in the risk of OHE of 8%. ANT, animal naming test; OHE, overt hepatic encephalopathy; TIPS, transjugular intrahepatic portosystemic shunt.

Baseline characteristics of the patients are provided in Table S1, for the whole cohort, and according to the predefined cut-off value of ANT of 20/minute for MHE in France. Patients with an ANT <20/min on the day of TIPS placement were significantly older (62 *vs.* 58 years, *p* = 0.01) and had poorer liver function, as assessed by Child-Pugh (8 *vs*. 7, *p* <0.001) or MELD (12 *vs.* 10, *p* = 0.04) score. They were more frequently treated with lactulose before TIPS (51 *vs*. 26%, *p* = 0.02). The median duration of hospitalization was 3.5 days, and only 13 patients were discharged more than 7 days after TIPS. During a median follow-up of 364 days (IQR 54–754 days), 30 patients (30%) developed an OHE episode, and 26 patients (26%) died or were transplanted. Fig. 1A displays the cumulative incidence of OHE (considering LT and death as competing risks) after TIPS. Univariable and multivariable analyses of factors associated with subsequent development of OHE are provided in Table S2A and B: older age (subdistribution hazard ratio [sHR] 1.05; 95% CI 1.00–1.10; *p* = 0.048), a higher baseline ammonia level before TIPS (sHR 1.01; 95% CI 1.01–1.01; *p* <0.001), a lower ANT at discharge (sHR 0.91; 95% CI 0.83–0.99; *p* = 0.032), and a higher ammonia level at discharge (sHR 1.02; 95% CI 1.01–1.03; *p* = 0.04) were independently associated with OHE after TIPS, but not ANT on the day of TIPS placement (sHR 0.96; 95% CI 0.90–1.03; *p* = 0.06). The evolution of ANT between the day of TIPS, discharge, and 1, 3, and 6 months after TIPS is provided in Fig. 1B. The relation between ANT at any time after TIPS placement and the HR of post-TIPS OHE is shown in Fig. 1C. A proportionality test revealed that the prognostic value of post-TIPS ANT was stable over time. Each decrease of ANT of 1 point was associated with an increased risk of OHE of 8%.

Cumulative mortality (LT as a competing event) was 26% at 1 year after TIPS. ANT, before or at each time point after TIPS, was never associated with a higher risk of death or LT (HR 0.92; 95% CI 0.82-1.03; *p* = 0.13).

## Discussion

In this study, we confirm that ANT, evaluated before elective TIPS placement, is not associated with subsequent development of OHE. However, we suggest for the first time that assessment of this test at discharge – which varied among patients but occurred a median of 3.5 days after elective TIPS placement in our study – may be a useful tool for predicting OHE. Second, ammonia levels before TIPS could be a good biomarker for identifying patients at risk of OHE after TIPS. Recent studies have demonstrated the prognostic value of ammonia, both in outpatients (predicting hospitalization, OHE, and death)^17^ and in patients hospitalized for acute decompensation (predicting acute-on-chronic liver failure and death).^18^ Our team also conducted a recent study comparing the prevalence and risk factors of OHE after elective or preemptive TIPS and found that baseline ammonia was an independent factor associated with OHE after elective but not after preemptive TIPS, although baseline ammonia was significantly higher in the preemptive TIPS group.^19^ In a recent report by Labenz *et al.*,^16^ ammonia measured after TIPS insertion was found to be useful for identifying patients at risk for OHE. The authors hypothesized that if ammonia homeostasis is dramatically changed by TIPS insertion, the severity of the change in ammonia metabolism is not predictable. Our results align with these findings, suggesting that early changes after TIPS (in ANT or ammonia levels) may have an early impact on brain function, act as an initial cerebral insult, and contribute to early cognitive impairment detected by ANT after TIPS, which is predictive of OHE. Interestingly, both ANT and ammonia are linked to further OHE, suggesting that the ANT might be sensitive to changes in ammonia levels and to causes of neurocognitive impairment other than covert HE, as previously described in outpatients with cirrhosis and cognitive complaints.^20^

In conclusion, our study provides evidence that ANT assessed at discharge may help identify patients at high risk of post-TIPS OHE. This easily obtainable measure could contribute to more tailored management of high-risk patients from a neurological perspective, such as intensified surveillance and treatment based on discharge ANT, avoidance of additional cerebral risk factors, patient education, and early consideration of shunt reduction and/or LT.

## Abbreviations

ANT, animal naming test; LT, liver transplantation; MELD, model for end stage liver disease; MHE, minimal hepatic encephalopathy; OHE, overt hepatic encephalopathy; TIPS, transjugular intrahepatic portosystemic shunt.

## Authors’ contributions

Melisande Jorus: collecting data, analysis and interpretation of data, drafting the manuscript. Philippe Sultanik: statistical analysis, critical review of the manuscript. Charlotte Bouzbib: management of patients, collecting data critical review of manuscript. Sarah Mouri: management of patients, collecting data critical review of manuscript. Lyes Kheloufi: critical review of manuscript. Nicolas Weiss: critical review of manuscript. Dominique Thabut: study design, management of patients, critical review of manuscript. Marika Rudler: study design, management of patients, analysis and interpretation of data, drafting the manuscript, critical review of manuscript.

## Data availability

Data available on justified request.

## Financial support

No financial support was received to produce this manuscript.

## Conflict of interest

The authors declare no conflicts of interest pertaining to this manuscript.

Please refer to the accompanying ICMJE disclosure forms for further details.
