# Supplementary material for: Animal naming test at discharge is associated with hepatic encephalopathy after elective TIPS
Source: JHEP Rep. 2025 Nov 29;8(3):101701. doi: 10.1016/j.jhepr.2025.101701 (PMC12890701; doi:10.1016/j.jhepr.2025.101701)
Supplement: Multimedia component 1 [file mmc1.pdf]

Animal naming test at discharge is associated with hepatic encephalopathy after elective TIPS

Melisande Jorus, Philippe Sultanik, Charlotte Bouzbib, Sarah Mouri, Lyes Kheloufi, Maxime Gasperment, Nicolas Weiss, Charles Roux, Dominique Thabut, Marika Rudler

Table of contents

Supplementary methods…………………………………………………………......2

Table S1…………………………………………………………………………......4

Table S2…………………………………………………………………………......6

Supplementary methods

More than 20 procedures were performed each year in the center, except in 2020 due to Covid-19 pandemic. All patients underwent TIPS placement using volume-controlled 8 or 10mm stents (W.L. Gore SRL, Flagstaff, AZ) as previously described (5), dilated to 8 or 10 mm, according to hemodynamic response. The aim was to reduce portal pressure gradient (PPG) below 12 mmHg. Hepatic venous pressure gradient and PPG were evaluated before and immediately after TIPS placement, respectively, under general anesthesia. Systematic embolization of shunts, before or after TIPS placement was not performed in our center.

Testing for covert hepatic encephalopathy (CHE) was done on the day of TIPS placement using the ANT test at 1 minute. A score <20 was considered pathologic at baseline. After discharge, each patient was followed-up at 1 month and then regularly (each 3 months or more if needed) by a hepatologist of the hepato-gastroenterology department of La Pitié-Salpêtrière hospital. Follow-up data (occurrence of OHE (including asterixis alone), ANT (as a continuous value) , death, liver transplantation) were collected at each consultation. OHE was diagnosed following the European Association for the Study of the Liver Practice guidelines (21), and was graded according to the West Haven (WH) classification, OHE defined as West-Haven grade 2-4. Patients with isolated asterixis were considered to have grade 2 OHE, according to the guidelines.

Data were analyzed using R 4.3.3, RStudio version 2023.12.1.402 and GraphPad Prism Version 8.0.2. We present categorical data as numbers with percentages and continuous data as median with interquartile range (IQR). Pairwise comparisons between patients with ammonia levels above or below the ULN were performed with the χ^2 test, Wilcoxon rank sum test, or the Fisher exact test, as appropriate. Comparisons between paired groups (longitudinal ammonia levels) were performed using Friedman's test.

The {tidycmprsk} R package was used for both cumulative incidence functions for competing risk analyses, and Fine and Gray competing risk regression analyses. The coding for the multi-state models with 3 states for OHE development analysis was: 0: alive without liver transplantation and no OHE event at the end of follow-up; 1: OHE event during follow-up; and 2: death or liver transplantation without prior OHE event during follow-up. This means that death or liver transplantation prior to an OHE event

were treated as competing events. Iterative ANT measures during study (at admission, at TIPS, at discharge and during follow-up) were incorporated in a time dependent Cox model to evaluate the impact of ANT as a continuous variable during follow-up on OHE development. For each Proportionalised test with Schoenfeld test was used to assert accuracy of the Cox model.

Table S1: Baseline characteristics according to the animal naming test on the day of TIPS placement

Variables	Whole population n=100	ANT before TIPS ≥ 20 /min n = 57	ANT before TIPS < 20 /min n = 43	p
**Age (yrs)**	59 (51-66)	58 (51 – 64)	62 (55 – 67)	**0.01**
**Male gender** *n (%)*	80 (80)	47 (82)	33 (76)	0.61
**BMI (kg/m2)**	26 (22-28)	25 (22 – 29)	25 (23 – 28)	0.89
**Obesity, overweight** *n (%)*	55 (55)	27 (57%)	22 (53%)	0.28
**Diabetes** *n (%)*	37 (37)	18 (42%)	17 (46%)	0.57
**Hypertension** *n (%)*	34 (34)	16 (34%)	18 (43%)	0.45
**Previous OHE** *n (%)*	24 (24)	13 (23)	11 (26)	0.93
**Previous AVB** *n (%)*	40 (40)	18 (38%)	15 (38%)	0.62
**Previous HCC** *n (%)*	7 (7)	3 (7%)	3 (9%)	0.70
**Child-Pugh class**				0.07
**A** *n (%)*	16	12 (25%)	4 (9,7%)	
**B** *n (%)*	77	34 (72%)	33 (80%)	
**C** *n (%)*	5	1 (2%)	4 (9%)	
**Child-Pugh score**	8 (7-8)	7 (6 – 8)	8 (8 – 9)	**<0.001**
**MELD score**	12 (9-14)	10 (9 – 12)	12 (10 – 14)	**0.04**
**Cause of cirrhosis** *n (%)*				0.94
**ALD** *n (%)*	39 (39)	19 (40%)	17 (41%)	
**MetALD** *n (%)*	30 (30)	11 (23%)	10 (25%)	
**MASLD** *n (%)*	14 (14)	7 (15%)	7 (17%)	
**Other** *n (%)*	17 (17)	10 (22%)	7 (17%)	
**TIPS indication** *N (%)*				0.06
**Ascites**	63 (63)	28 (59%)	26 (63%)	
**Secondary prophylaxis of AVB**	19 (19)	8 (17%)	9 (22%)	
**Hydrothorax**	5 (5)	1 (2%)	4 (9%)	
**Before surgery**	13 (13)	10 (21%)	2 (4%)	
**Haemoglobin (g/dl)**	11 (9-12)	11 (10 – 13)	10 (8 –12)	0.29
**Platelet count (G/L)**	111 82-164)	106 (81 – 168)	107 (70 – 144)	0.19
**PT (%)**	65 (57-71)	69 (61 – 76)	59 (51 – 67)	**<0.001**
**INR**	1.3 (1.2-1.5)	1,3 (1,2 – 1,4)	1,4 (1,3 – 1,6)	**<0.001**
**Serum sodium (mmol/L)**	135 (132-138)	136 (130 – 138)	133 (131 – 138)	0.35
**AST (UI/L)**	40 (30-57)	37 (27 – 49)	39 (30 – 55)	<0.90
**ALT (UI/L)**	23 (16-36)	22 (14 – 34)	23 (16 – 33)	0.67
**Bilirubin (µmol/L)**	18 (10-27)	15 (10 – 24)	21 (14 – 31)	0.04
**Albumin (g/L)**	32 (28-34)	32 (30 – 36)	30 (27 – 33)	**<0.001**
**Creatinine (µmol/L)**	82 (64-100)	80 (65 – 99)	84 (65 – 102)	0.46
**Ammonia (µmol/L)**	39 (30-55)	38 (15 – 55)	40 (30 – 56)	0.51
**Lactulose** *N (%)*	27 (27)	15 (26)	22 (51)	**0.02**

**Rifaximin** *N (%)*	78 (78)	47 (82%)	31 (72%)	0.23

Values are expressed as mean, median or absolute value (%). Student's t test was used for group comparisons of normally distributed continuous variables. Group comparisons of categorical variables were performed using Chi-squared test. A p value <0.05 was considered significant.

Abbreviations

BMI, body mass index; MELD, Model For End-Stage Liver Disease; MASLD, Metabolic-dysfunction Associated Steatotic Liver Disease; ALD, Alcohol related liver disease; MetALD, Metabolic and Alcohol related Liver Disease; AVB, acute variceal bleeding; OHE, overt hepatic encephalopathy; HCC, hepatocellular carcinoma; PT, prothrombin time ratio; INR, international normalized ratio; AST aspartate aminotransferase; ALT, alanine aminotransferase;

Table S2a: univariable analysis of factors associated with OHE development after TIPS

Variable	sHazard Ratio (CI 95%)	p
**Age**	1.05 (1.01 – 1.10)	**0.015**
**Previous OHE**	1.09 (0.46 – 2.57)	0.84
**Lactulose at TIPS placement**	1.28 (0.62 – 2.64)	0.50
**Rifaximin at TIPS placement**	0.94 (0.41 – 2.20)	0.90
**Platelet count**	1.00 (1.00 – 1.00)	0.03
**MELD score**	1.06 (0.98 – 1.15)	0.14
**PT**	0.98 (0.96 – 1.01)	0.14
**INR**	2.63 (0.77 – 8.94)	0.12
**Bilirubin**	1.01 (1.00 – 1.03)	0.18
**Creatinine**	1.00 (0.99 – 1.00)	0.61
**Albumin**	0.87 (0.80 – 0.96)	**0.004**
**Ammonia at baseline**	1.01 (1.01 – 1.02)	**<0.001**
**ANT on the day of TIPS placement**	0.93 (0.88 – 0.97)	**0.002**
**ANT at discharge**	0.88 (0.86 – 1.01)	**0.001**
**Ammonia at discharge**	1.01 (1.00 – 1.02)	**0.02**

OHE, overt hepatic encephalopathy; MELD, Model For End-Stage Liver Disease; PT, prothrombin time ratio; INR, international normalized ratio; ANT, animal naming test

Table S2b: multivariable analysis of factors associated with OHE development after TIPS

Model 1

Variable	sHazard Ratio (CI 95%)	p
**Age**	1.05 (1.00 – 1.10)	**0.048**
**MELD score**	1.00 (0.99 – 1.21)	0.068
**Ammonia**	1.02 (1.01 – 1.02)	**<0.001**
**ANT on the day of TIPS placement**	0.96 (0.91 – 1.02)	0.2

MELD, Model For End-Stage Liver Disease; ANT, animal naming test

Model 2

Variable	sHazard Ratio (CI 95%)	p
**Age**	1.05 (1.00 – 1.10)	**0.035**
**MELD score**	1.15 (1.04 – 1.28)	**0.005**
**Ammonia**	1.01 (1.00 – 1.02)	**0.036**
**ANT at discharge**	0.91 (0.83 – 0.99)	**0.032**

MELD, Model For End-Stage Liver Disease; ANT, animal naming test

Model 3

Variable	sHazard Ratio (CI 95%)	p
**ANT evolution**	0.92 (1.00 – 1.10)	**0.002**
**MELD score**	1.10 (1.04 – 1.28)	**0.02**
**Ammonia**	1.01 (1.00 – 1.02)	**0.03**

MELD, Model For End-Stage Liver Disease; ANT, animal naming test

Model 4

Variable	sHazard Ratio (CI 95%)	p
**ANT at discharge**	0.93 (0.87 – 0.99)	**0.04**
**Age**	1.04 (0.99 – 1.09)	0.09
**Ammonia at discharge**	1.01 (1.00 – 1.02)	**0.03**

ANT, animal naming test
